# Establishment and validation of a nomogram to predict the in-hospital death risk of nosocomial infections in cancer patients

**DOI:** 10.1186/s13756-022-01073-3

**Published:** 2022-02-07

**Authors:** Aimin Jiang, Xin Shi, Haoran Zheng, Na Liu, Shu Chen, Huan Gao, Mengdi Ren, Xiaoqiang Zheng, Xiao Fu, Xuan Liang, Zhiping Ruan, Tao Tian, Yu Yao

**Affiliations:** 1 Department of Medical Oncology, The First Affiliated Hospital of Xi’an Jiaotong University, No. 277 Yanta West Road, Xi’an, 710061 Shaanxi People’s Republic of China; 2 School of Public Health, Xi’an Jiaotong University Health Science Center, No. 76 Yanta West Road, Xi’an, 710061 Shaanxi People’s Republic of China; 3 Department of Hematology, The First Affiliated Hospital of Xi’an Jiaotong University, No. 277 Yanta West Road, Xi’an, 710061 Shaanxi People’s Republic of China

**Keywords:** Cancer patients, Nosocomial infections, Microbiological distribution, Antimicrobial susceptibility, Mortality

## Abstract

**Background:**

Attributed to the immunosuppression caused by malignancy itself and its treatments, cancer patients are vulnerable to developing nosocomial infections. This study aimed to develop a nomogram to predict the in-hospital death risk of these patients.

**Methods:**

This retrospective study was conducted at a medical center in Northwestern China. The univariate and multivariate logistic regression analyses were adopted to identify predictive factors for in-hospital mortality of nosocomial infections in cancer patients. A nomogram was developed to predict the in-hospital mortality of each patient, with receiver operating characteristic curves and calibration curves being generated to assess its predictive ability. Furthermore, decision curve analysis (DCA) was also performed to estimate the clinical utility of the nomogram.

**Results:**

A total of 1,008 nosocomial infection episodes were recognized from 14,695 cancer patients. Extended-spectrum β-lactamase (ESBL)-producing *Escherichia coli* (15.5%) was the most predominant causative pathogen. Besides, multidrug-resistant strains were discovered in 25.5% of cases. The multivariate analysis indicated that Eastern Cooperative Oncology Group Performance Status 3–4, mechanical ventilation, septic shock, hypoproteinemia, and length of antimicrobial treatment < 7 days were correlated with higher in-hospital mortality. Patients who received curative surgery were correlated with favorable survival outcomes. Ultimately, a nomogram was constructed to predict the in-hospital mortality of nosocomial infections in cancer patients. The area under the curve values of the nomogram were 0.811 and 0.795 in the training and validation cohorts. The calibration curve showed high consistency between the actual and predicted in-hospital mortality. DCA indicated that the nomogram was of good clinical utility and more credible net clinical benefits in predicting in-hospital mortality.

**Conclusions:**

Nosocomial infections stay conjoint in cancer patients, with gram-negative bacteria being the most frequent causative pathogens. We developed and verified a nomogram that could effectively predict the in-hospital death risk of nosocomial infections among these patients. Precise management of high-risk patients, early recognition of septic shock, rapid and adequate antimicrobial treatment, and dynamic monitoring of serum albumin levels may improve the prognosis of these individuals.

**Supplementary Information:**

The online version contains supplementary material available at 10.1186/s13756-022-01073-3.

## Introduction

Currently, malignant tumors have become one of the major public health problems that threaten human health worldwide. According to cancer statistics, in 2020, there will be 19.3 million newly diagnosed cancer cases and 10 million death cases all over the world [1]. No matter cancer incidence or mortality, solid tumors are at the forefront. Although significant advances in the treatment of malignancy have been made over the past few decades, the prognosis of these patients remains bleak. Besides, cancer patients are more vulnerable to developing severe infection owing to the malignancy itself and its treatments, especially surgery and cytotoxic therapies [2]. As a result, it delays the initiation of chemotherapy and reduces its relative dosage intensity (RDI). At the same time, it also prolongs hospitalization and increases the healthcare burden [3, 4]. Therefore, it is pivotal for clinicians to fully understand the local epidemiological and microbiological characteristics of nosocomial infections. It also can play an essential role in reducing the mortality of these individuals [2].

To date, a growing number of studies have focused on the bacteriological characteristics and antibiotic resistance pattern of nosocomial infections in patients with malignancy. However, most studies only concentrate on bloodstream infections (BSIs), despite other infections being more common than BSIs in these patients (such as respiratory tract and urinary tract infections). Furthermore, numerous studies reported that the microbial distribution and antibiotic sensitivity of cancer patients who developed nosocomial infections might be influenced by the source of infection [5, 6]. Moreover, several previous studies have found that cancer patients with nosocomial infections were highly resistant to empirical antibiotic therapy due to the phenomenon of multidrug-resistant (MDR) [5, 7]. Importantly, although many studies have evaluated the clinical characteristics and prognostic factors of nosocomial infections in cancer patients, most studies enrolled a small number of sample size patients and drew inconclusive conclusions. Meanwhile, no study comprehensively investigated the clinical features of nosocomial infections in cancer patients and developed a predictive model to predict their in-hospital mortality. In this premier, we implemented this study to investigate the clinical features of nosocomial infections in patients with solid tumors and develop a nomogram to predict the in-hospital mortality of these patients.

## Methods and materials

### Study design

This single-center retrospective cohort study was performed in the First Affiliated Hospital of Xi’an Jiaotong University, the medical center in Northwestern China. The oncology center in this hospital was composed of three departments (medical oncology ward, surgical oncology ward, and radiotherapy oncology ward). The electronic medical charts of all nosocomial infection cases hospitalized in the oncology center from August 2013 to May 2019 were retrospectively reviewed. All solid tumors were confirmed through histological or cytological pathology. All cancer patients identified with nosocomial infections during hospitalization were included in the present study if they were older than 18 years old. Patients under the age of 18 years old or without complete medical records were excluded. This research is approved by the local ethics committee of the First Affiliated Hospital of Xi’an Jiaotong University.

### Data collection

Three investigators (AJ, HZ, and NL) extracted eligible patients' clinical data and laboratory parameters from electronic medical charts. The detailed clinical data were as follows: gender, age, smoking history, Eastern Cooperative Oncology Group performance status (ECOG-PS), the underlying malignancy type, stage of cancer, source of infection, comorbidities, the degree of fever, the types of anticancer treatment within one-month (surgery, chemotherapy, or radiotherapy), the regimen of empirical antibiotic treatment, length of antimicrobial therapy, the presence of indwelling catheters, the existence of septic shock during hospitalization, the use of a ventilator, and intensive care unit (ICU) admission during the hospitalization. Laboratory parameters of each patient were also reviewed, including the results of blood routine tests and serum albumin level. Furthermore, we collected the results of drug susceptibility tests of the isolated pathogens to commonly used antibiotics.

### Definitions

Hospitalized cancer patients were considered as nosocomial infection cases if they met the following criteria [8]: (a) on the premise of excluding contamination of clinical specimens, the results of microbial culture indicated that at least one pathogen was positive (> 48 h after hospital admission); (b) there were corresponding clinical manifestations, laboratory examination results, or radiological results recorded which is in electronic medical records; or (c) a clear infection type record that is acquired from the electronic medical records. The diagnosis of nosocomial respiratory tract infection was mainly based on microorganism culture from respiratory secretions, sputum, and or/ bronchoalveolar lavage fluid (BALF). Radiological findings of new or progressive infiltrate, consolidation, and effusion were also essential diagnostic conditions [8]. Besides, patients’ symptoms, signs, and blood tests were also needed to be considered. The final diagnosis needs to be assessed by qualified physicians, and the radiologist was also needed to be consulted when necessary. Nosocomial urinary tract infection was considered by positive urine culture (> 10^4^ colony-forming units/mL of no more than two different species of microorganisms) obtained at least 48 h after hospital admission [8, 9]. BSI was defined by a positive blood culture obtained from a patient at the time of hospital admission or within 48 h of admission, as previously described [10]. All BSIs cases in this study are hospital-acquired.

The shock was referred to as systolic blood pressure < 90 mmHg, and fluid therapy and/or vasoactive medications have no improvement on this condition. Fever was considered an axillary temperature of 38.3 °C on one instance or a temperature of > 38.0 °C on two or more occasions throughout 12 h [11, 12]. Hypoproteinemia was defined as the level of serum albumin < 30 g/L.

MDR strains were defined based on the previous descriptions [13, 14]. Antimicrobial susceptibility testing was performed using the Kirby-Bauer disc diffusion method under the requirement of the Clinical and Laboratory Standards Institute (CLSI) guidelines [15].

### Study outcome and cohort establishment

In this study, in-hospital mortality was used as the primary study outcome. Only death cases that were caused by nosocomial infections during hospitalization were selected for in-hospital mortality calculation. Nevertheless, death cases unrelated to nosocomial infections (such as malignancy itself or other complications) were not considered for in-hospital mortality calculation.

All eligible patients enrolled in the whole dataset were randomly divided into training and validation cohorts according to a ratio of 7:3 by exploiting the “createDataPartition” function in R software. The current study used the training cohort to develop a nomogram to predict the in-hospital death risk. The validation cohort was adopted to verify its predictive ability and clinical utility.

### Statistical analysis

The continuous variables were summarized as means and standard deviations (SD) or medians and interquartile (IQR) as appropriate, while categorical variables were presented as frequency and percentage. The difference between categorical variables was compared using Chi-square or Fisher’s exact tests, while a two-independent sample t-test or Mann–Whitney U test was used to compare the difference between continuous variables. Univariate and multivariate logistic regression analyses were adopted to investigate the independent risk factors for in-hospital mortality of nosocomial infections. Variables with a *P* value < 0.05 in the multivariate regression analysis were selected to construct a nomogram using R software, “rms” and “regplot” packages to calculate each patient's in-hospital death risk. Furthermore, the receiver operating characteristic (ROC) curves and calibration curves were used to assess the predictive ability of the nomogram. Decision curve analysis (DCA) was also performed via R software, “DecisionCurve” package to estimate the clinical utility and net clinical benefits of the nomogram when it was adopted to support the clinical practice. All statistical analyses were conducted using the SPSS software version 22.0 and R software version 3.6.3.

## Results

### Demographical characteristics of the participants

**Fig. 1:**
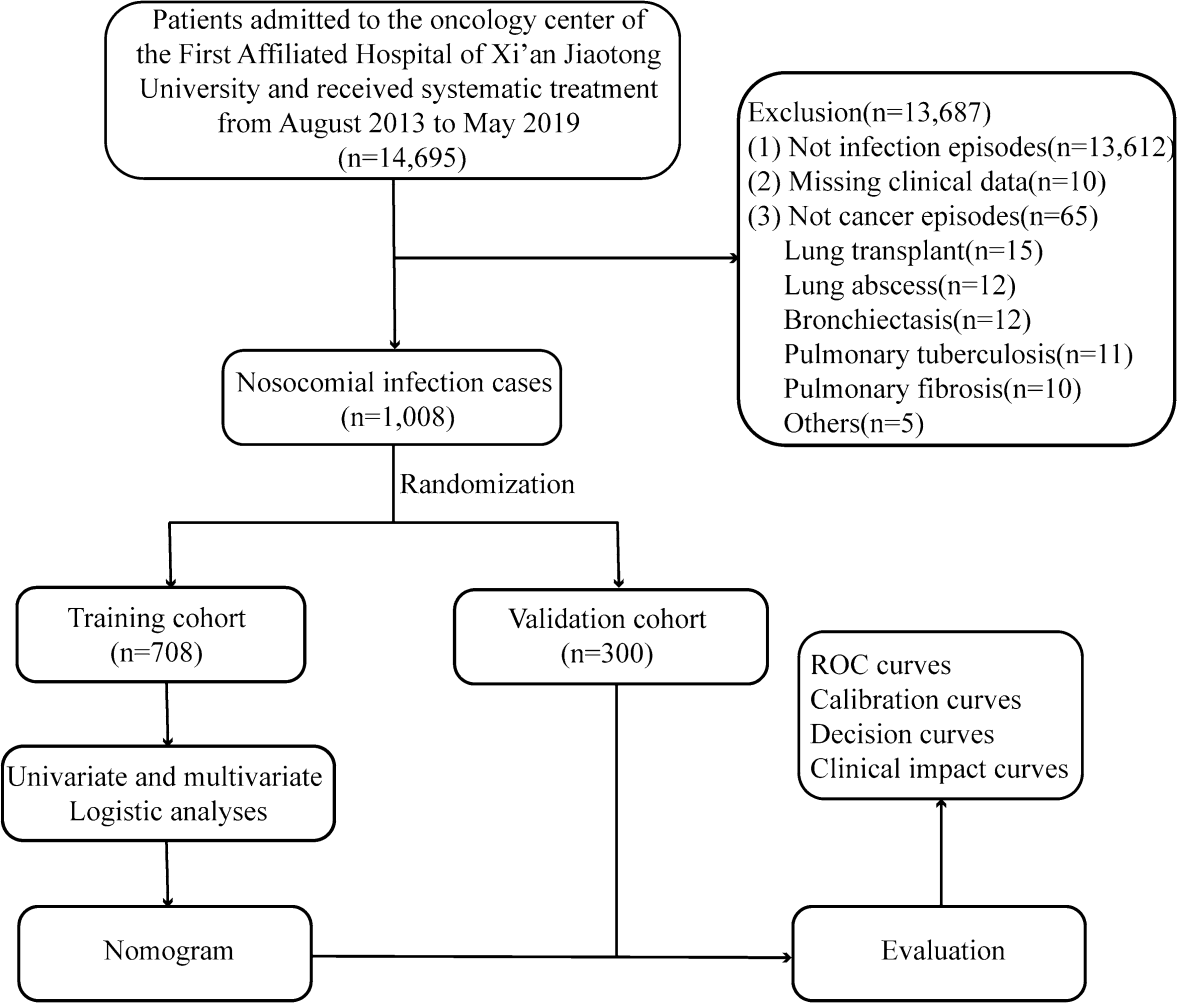
Flow chart to investigate the clinical characteristics of nosocomial infections among cancer patients and establish a nomogram for in-hospital death risk prediction

During the study period, a total of 14,695 cancer patients were admitted to the oncology departments of the First Affiliated Hospital of Xi’an Jiaotong University. After excluding 13,612 cases of non-nosocomial infection, 10 cases without complete electronic medical records, and 65 cases diagnosed with benign disease, a total of 1,008 participants developed nosocomial infections during the period (Fig. 1). Among them, 563 were males (55.9%) and 445 were females (44.1%), with a median age of 61 years old (range: 54 to 68). Upper gastrointestinal tract tumor was the most common type of malignancy (285 cases, 28.3%), followed by gynecological tumor (211 cases, 20.9%) and lung cancer (193 cases, 19.1%). Most of the patients (951 cases, 94.3%) had ECOG-PS less than 3, and 603 (59.8%) of the cases were diagnosed at an advanced stage (stage III-IV). Table 1 presented the detailed demographical characteristics of participants.

**Table 1:** Demographic and clinical characteristics of nosocomial infections in cancer patients

Variables	Whole cohort (n = 1008)	Training cohort (n = 708)			Validation cohort (n = 300)	*P*^b^
		Survival (n = 610)	Death (n = 98)	*P*^a^		
Sex (male)	563 (55.9)	336 (55.1)	63 (64.3)	0.088	164 (54.7)	0.621
Age (years old)	61 (54–68)	61 (54–69)	64 (54–71)	0.209	60 (53–67)	0.015*
≥ 61	533 (52.9)	332 (54.4)	60 (61.2)		141 (47.0)	
< 61	475 (47.1)	278 (45.6)	38 (38.8)		159 (53.0)	
Tobacco use	430 (42.7)	261 (42.8)	45 (45.9)	0.561	124 (41.3)	0.580
ECOG-PS				< 0.001*		0.992
0–2	951 (94.3)	591 (96.9)	77 (78.6)		283 (94.3)	
3–4	57 (5.7)	19 (3.1)	21 (21.4)		17 (5.7)	
Underlying malignancy type						
Head and neck cancer	34 (3.4)	16 (2.6)	8 (8.2)	0.012*	10 (3.3)	0.964
Lung cancer	193 (19.1)	106 (17.4)	32 (32.7)	< 0.001*	55 (18.3)	0.669
Upper gastrointestinal cancer	285 (28.3)	180 (29.5)	23 (23.5)	0.220	82 (27.3)	0.666
Hepatobiliary and pancreatic cancer	52 (5.2)	30 (4.9)	7 (7.1)	0.358	15 (5.0)	0.882
Breast cancer	94 (9.3)	67 (11.0)	6 (6.1)	0.142	21 (7.0)	0.098
Colon and rectal cancer	91 (9.0)	55 (9.0)	9 (9.2)	0.957	27 (9.0)	0.984
Genitourinary cancer	48 (4.8)	33 (5.4)	3 (3.1)	0.463	12 (4.0)	0.460
Gynecological cancer	211 (20.9)	123 (20.2)	10 (10.2)	0.019*	78 (26.0)	0.010*
Stage of cancer				0.045*		0.729
Stage I–II	405 (40.2)	252 (41.3)	30 (30.6)		123 (41.0)	
Stage III–IV	603 (59.8)	358 (58.7)	68 (69.4)		177 (59.0)	
Comorbidities						
COPD	48 (4.8)	23 (3.8)	10 (10.2)	0.011*	15 (5.0)	0.817
Liver disease	53 (5.3)	29 (4.8)	13 (13.3)	0.001*	11 (3.7)	0.141
Diabetes mellitus	99 (9.8)	50 (8.2)	11 (11.2)	0.321	38 (12.7)	0.048*
Renal disease	42 (4.2)	22 (3.6)	11 (11.2)	0.002*	9 (3.0)	0.228
Source of infection						
Respiratory tract	376 (37.3)	227 (37.2)	46 (46.9)	0.066	103 (34.3)	0.205
Gastrointestinal	36 (3.6)	16 (2.6)	7 (7.1)	0.019*	13 (4.3)	0.396
Urinary tract	250 (24.8)	159 (26.1)	13 (13.3)	0.006*	78 (26.0)	0.566
Skin and soft tissue	98 (9.7)	69 (11.3)	2 (2.0)	0.005*	27 (9.0)	0.614
Thoracic cavity	59 (5.9)	35 (5.7)	2 (2.0)	0.127	22 (7.3)	0.193
Abdominal cavity	59 (5.9)	36 (5.9)	5 (5.1)	0.753	18 (6.0)	0.897
BSI	130 (12.9)	68 (11.1)	23 (23.5)	0.001*	39 (13.0)	0.949
Existence of fever	403 (40.0)	229 (37.5)	54 (55.1)	0.001*	120 (40.0)	0.993
Previous surgery (within 1 month)				< 0.001*		0.299
None	668 (66.3)	387 (63.4)	87 (88.8)		194 (64.7)	
Curative surgery	314 (31.2)	205 (33.6)	8 (8.2)		101 (33.7)	
Palliative surgery	26 (2.6)	18 (3.0)	3 (3.1)		5 (1.7)	
Previous chemotherapy (within 1 month)				0.245		0.950
None	625 (62.0)	371 (60.8)	70 (71.4)		184 (61.3)	
Neoadjuvant	12 (1.2)	8 (1.3)	1 (1.0)		3 (1.0)	
Adjuvant	198 (19.6)	126 (20.7)	14 (14.3)		58 (19.3)	
1st line	114 (11.3)	71 (11.6)	6 (6.1)		37 (12.3)	
2nd line	42 (4.2)	23 (3.8)	5 (5.1)		14 (4.7)	
≥ 3rd line	17 (1.7)	11 (1.8)	2 (2.0)		4 (1.3)	
Previous radiotherapy (within 1 month)	142 (14.1)	89 (14.6)	9 (9.2)	0.150	44 (14.7)	0.731
Empirical antibiotic treatment						
β-lactam/β-lactamase inhibitor	161 (16.0)	92 (15.1)	11 (11.2)	0.315	58 (19.3)	0.058
Third-generation cephalosporins	133 (13.2)	80 (13.1)	10 (10.2)	0.422	43 (14.3)	0.487
Carbapenems	24 (2.4)	12 (2.0)	1 (1.0)	0.808	11 (3.7)	0.081
Fluoroquinolones	117 (11.6)	71 (11.6)	9 (9.2)	0.476	37 (12.3)	0.639
Aminoglycosides	8 (0.8)	3 (0.5)	0 (0.0)	1.000	5 (1.7)	0.100
Combination therapy	405 (40.2)	250 (41.0)	55 (56.1)	0.005*	100 (33.3)	0.004*
Length of antimicrobial therapy (day)	7 (4–11)	7 (4–11)	6 (3–10)	0.016*	7 (4–10)	0.546
≥ 7	563 (56.4)	359 (50.7)	45 (6.4)		165 (55.0)	
< 7	439 (43.6)	251 (35.5)	53 (7.5)		135 (45.0)	
Presence of indwelling catheters	564 (56.0)	350 (57.4)	45 (45.9)	0.034*	169 (56.3)	0.874
ICU admission	99 (9.8)	68 (11.1)	13 (13.3)	0.541	18 (6.0)	0.008*
Mechanical ventilation	66 (6.5)	35 (5.7)	14 (14.3)	0.002*	17 (5.7)	0.462
Septic shock	124 (12.3)	58 (9.5)	37 (37.8)	< 0.001*	29 (9.7)	0.097
Laboratory examination results						
Hemoglobin (g/L; normal range 115–150)	108.2 ± 20.1	108.9 ± 19.5	103.4 ± 22.3	0.010	108.3 ± 20.56	0.921
< 110	536 (53.2)	325 (53.3)	61 (62.2)	0.098	150 (50.0)	0.189
Platelet count (× 10^9^/L; normal range 125–350)	211.1 ± 113.4	217.8 ± 117.8	172.9 ± 113.7	< 0.001	210.1 ± 101.6	0.852
< 100.0	131 (13.0)	67 (11.0)	25 (25.5)	< 0.001*	39 (13.0)	0.998
White-cell count (× 10^9^/L; normal range 4.0–10.0)	8.1 ± 5.1	8.2 ± 5.1	9.1 ± 6.8	0.257	7.6 ± 4.2	0.021*
> 10.0	265 (26.3)	168 (27.5)	34 (34.7)	0.146	63 (21.0)	0.013*
< 4.0	161 (16.0)	93 (15.2)	20 (20.4)	0.195	48 (16.0)	0.987
Neutrophils count (× 10^9^/L)	6.6 ± 4.8	6.6 ± 4.9	7.7 ± 6.4	0.107	6.1 ± 4.0	0.026*
Lymphocytes count (× 10^9^/L; normal range 1.1–3.2)	1.0 ± 0.6	1.0 ± 0.6	0.8 ± 0.6	0.009	1.0 ± 0.6	0.800
< 1.0	583 (57.8)	352 (57.7)	71 (72.4)	0.006*	160 (53.3)	0.059
Albumin (g/L; normal range 40–55)	34.2 ± 6.1	34.6 ± 5.8	30.9 ± 6.1	< 0.001	34.5 ± 6.2	0.303
< 30.0	263 (26.1)	140 (23.0)	49 (50.0)	< 0.001*	74 (24.7)	0.503

ECOG-PS, Eastern Cooperative Oncology Group Performance Status; COPD, chronic obstructive pulmonary disease; BSI, bloodstream infection; ICU, intensive care unit

^a^*P* value for univariate analysis of the training cohort

^b^*P* value for clinical characteristics analysis between the training cohort and validation cohort

^*^Indicates statistical significance

### Infection-related characteristics of the participants

Subsequently, we also explored the infection-related characteristics of nosocomial infections in our study. Overall, 1008 (6.9%) cancer patients developed nosocomial infections during the period. Respiratory tract infection, urinary tract infection, and BSI were the most common types of nosocomial infection, accounting for 37.3%, 24.8%, and 12.9% of cases, respectively. In the current study, 84.1% of cases received empirical antibiotics therapy during hospitalization, with combination therapy being the most primary regimen (40.2%), followed by β-lactam/β-lactamase inhibitor (16.0%), third-generation cephalosporins (13.2%), and fluoroquinolones (11.6%). What’s more, 99 patients (9.8%) were admitted to the ICU during the study period, 66 patients (6.5%) received mechanical ventilation, and 124 patients (12.3%) experienced a septic shock (Table 1).

### Microbiological distribution characteristics and antimicrobial susceptibility analysis

We further analyzed the microbiological distribution characteristics of nosocomial infections among patients with solid tumors. We found that gram-negative bacteria were the main causative pathogens of nosocomial infections, accounting for 63.3% of all pathogenic bacteria, followed by gram-positive cocci (16.8%) and fungi (11.4%). In our study, there were 257 cases (25.5%) of nosocomial infections caused by MDR strains, with multi-drug resistant gram-negative bacilli (MDRGNB) being the most predominant pathogenic bacteria (24.8%). Overall, extended-spectrum β-lactamase (ESBL)-producing *Escherichia coli* (156 cases, 15.5%), ESBL-negative *E. coli* (135 cases, 13.4%), and *Pseudomonas aeruginosa* (86 cases, 8.5%) were the most common pathogens that caused nosocomial infections in cancer patients, as summarized in Table 2. We also investigated the microbiological distribution features between different medical wards and different malignancy types. We observed that gram-negative bacteria were the most frequently isolated pathogens, no matter in different medical wards or malignancy types (Additional file 1: Fig. S1). Subsequently, we investigated the drug sensitivity of the isolated organisms to commonly used antibiotics. The results indicated that the isolated gram-negative bacilli exhibited high sensitivity to amikacin (95.0%), meropenem (94.4%), imipenem (93.4%), and piperacillin/tazobactam (86.8%) (Additional file 1: Fig. S2A), while the isolated gram-positive cocci exhibited high sensitivity to teicoplanin (100.0%), vancomycin (100.0%), linezolid (100.0%), and tigecycline (100.0%) (Additional file 1: Fig. S2B).

**Table 2:** Causative pathogens of all nosocomial infection episodes in cancer patients

Causative organisms	Whole cohort (n = 1008)	Training cohort (n = 708)			Validation cohort (n = 300)	*P*^b^
		Survival (n = 610)	Death (n = 98)	*P*^a^		
Gram-negative bacteria	388 (38.5)	229 (37.5)	31 (31.6)	0.260	128 (42.7)	0.076
* Escherichia coli*	135 (13.4)	71 (11.6)	10 (10.2)	0.679	54 (18.0)	0.005*
* Klebsiella pneumoniae*	65 (6.4)	38 (6.2)	11 (11.2)	0.071	16 (5.3)	0.348
* Pseudomonas aeruginosa*	86 (8.5)	54 (8.9)	4 (4.1)	0.110	28 (9.3)	0.553
* Enterobacter spp.*	34 (3.4)	21 (3.4)	2 (2.0)	0.675	11 (3.7)	0.737
* Klebsiella oxytoca*	4 (0.4)	3 (0.5)	0 (0.0)	1.000	1 (0.3)	1.000
* Proteus mirabilis*	13 (1.3)	8 (1.3)	2 (2.0)	0.915	3 (1.0)	0.822
* Salmonella enterica serovar*	2 (0.2)	1 (0.2)	0 (0.0)	1.000	1 (0.3)	0.507
* Haemophilus spp*	42 (4.2)	28 (4.6)	2 (2.0)	0.372	12 (4.0)	0.863
* Serratia*	7 (0.7)	5 (0.8)	0 (0.0)	1.000	2 (0.7)	1.000
Gram-positive bacteria	143 (14.2)	87 (14.3)	14 (14.3)	0.995	42 (14.0)	0.912
* Staphylococcus aureus*	71 (7.0)	40 (6.6)	7 (7.1)	0.829	24 (8.0)	0.440
MRSA	7 (0.7)	4 (0.7)	2 (2.0)	0.196	1 (0.3)	0.681
* Streptococcus pneumoniae*	37 (3.7)	21 (3.4)	3 (3.1)	1.000	13 (4.3)	0.466
Coagulase-negative *staphylococci*	15 (1.5)	13 (2.1)	1 (1.0)	0.732	1 (0.3)	0.092
* Streptococcus anginosus*	13 (1.3)	9 (1.5)	1 (1.0)	1.000	3 (1.0)	0.822
Enterococcus	26 (2.6)	15 (2.5)	6 (6.1)	0.096	5 (1.7)	0.234
* E. faecalis*	12 (1.2)	7 (1.1)	3 (3.1)	0.303	2 (0.7)	0.496
* E. faecium*	10 (1.0)	5 (0.8)	2 (2.0)	0.251	3 (1.0)	1.000
* Enterococcus spp*	4 (0.4)	3 (0.5)	1 (1.0)	0.450	0 (0.0)	0.324
Anaerobes	6 (0.6)	5 (0.8)	0 (0.0)	1.000	1 (0.3)	0.676
Fungi	115 (11.4)	60 (9.8)	21 (21.4)	0.001	34 (11.3)	0.961
* Candida albicans*	79 (7.8)	44 (7.2)	13 (13.3)	0.041*	22 (7.3)	0.698
* Candida spp.*	14 (1.4)	7 (1.1)	2 (2.0)	0.805	5 (1.7)	0.844
* Aspergillus flavus*	22 (2.2)	9 (1.5)	6 (6.1)	0.010*	7 (2.3)	0.831
MDRGNB	250 (24.8)	162 (26.6)	23 (23.5)	0.518	65 (21.7)	0.134
ESBL-producing *Escherichia coli*	156 (15.5)	102 (16.7)	17 (17.3)	0.878	37 (12.3)	0.073
MDR *Pseudomonas aeruginosa*	13 (1.3)	10 (1.6)	0 (0.0)	0.415	3 (1.0)	0.822
* Acinetobacter baumannii*	30 (3.0)	18 (3.0)	1 (1.0)	0.447	11 (3.7)	0.401
* Stenotrophomonas maltophilia*	16 (1.6)	8 (1.3)	3 (3.1)	0.390	5 (1.7)	1.000
ESBL-producing *Klebsiella pneumoniae*	31 (3.1)	20 (3.3)	2 (2.0)	0.732	9 (3.0)	0.928
Carbapenem-resistant *Enterobacteriaceae*	4 (0.4)	4 (0.7)	0 (0.0)	1.000	0 (0.0)	0.324
Polymicrobial	80 (7.9)	52 (8.5)	3 (3.1)	0.061	25 (8.3)	0.762

MRSA, Methicillin-resistant *S. aureus*; MDRGNB, multidrug-resistant gram-negative bacilli; ESBL, extended-spectrum β-lactamase; MDR: multidrug-resistant

^a^*P* value for univariate analysis of the training cohort

^b^*P* value for clinical characteristics analysis between the training cohort and validation cohort

^*^Indicates statistical significance

### Predictive factors for in-hospital death risk of nosocomial infections in cancer patients

The overall case-fatality rate is 12.1% of nosocomial infections among cancer patients in our study. We compared the clinical characteristics, infection-related characteristics, and the distribution of causative pathogens of patients based on the survival outcomes during hospitalization in the training cohort to identify potential predictive factors of in-hospital mortality. The univariate logistic regression analysis indicated that several factors were correlated with the in-hospital mortality of nosocomial infections in these patients (*P* < 0.05, Tables 1 and 2). Subsequently, we performed multivariate analysis to identify the independent predictive factors of in-hospital death risk. Ultimately, we observed that ECOG-PS 3–4 [odds ratio (OR): 4.46, 95% confidence interval (CI) 2.04–9.74; *P* < 0.001], previous curative surgery (OR 0.18, 95% CI 0.07–0.52; *P* = 0.001), length of antimicrobial therapy < 7 days (OR 2.54, 95% CI 1.40–4.60; *P* = 0.002), mechanical ventilation (OR 4.17, 95% CI 1.42–12.23; *P* = 0.009), septic shock (OR 3.16, 95% CI 1.37–7.30; *P* = 0.007), and hypoproteinemia (OR 2.34, 95% CI 1.34–4.10; *P* = 0.003) were independent predictive factors of in-hospital death risk (Table 3).

**Table 3:** Multivariate logistic regression analysis for prognostic factors of nosocomial infections in cancer patients in the training cohort

Variables	β	SE	Wald	OR (95% CI)	*P*
ECOG-PS					
0–2					
3–4	1.49	0.40	14.02	4.46 (2.04–9.74)	< 0.001*
Underlying malignancy type					
Head and neck cancer	0.85	0.56	2.30	2.33 (0.78–6.98)	0.130
Lung cancer	0.26	0.33	0.62	1.30 (0.68–2.48)	0.431
Gynecological cancer	− 0.60	0.43	1.94	0.55 (0.24–1.28)	0.164
Stage of cancer	− 0.03	0.29	0.01	0.97 (0.55–1.71)	0.924
Source of infection					
Gastrointestinal tract	0.13	0.58	0.05	1.14 (0.36–3.59)	0.819
Urinary tract	− 0.54	0.42	1.63	0.59 (0.26–1.33)	0.202
Skin and soft tissue	− 1.25	0.79	2.50	0.29 (0.06–1.35)	0.114
BSI	− 0.66	0.46	2.06	0.52 (0.21–1.27)	0.152
Comorbidities					
COPD	0.14	0.54	0.06	1.15 (0.40–3.32)	0.803
Liver disease	0.80	0.44	3.39	2.23 (0.95–5.22)	0.066
Renal disease	0.86	0.50	2.95	2.36 (0.89–6.26)	0.086
Existence of fever	0.27	0.32	0.70	1.31 (0.70–2.45)	0.405
Previous surgery (within 1 month)					0.006*
None			10.21		
Curative surgery	− 1.69	0.53	10.20	0.18 (0.07–0.52)	0.001*
Palliative surgery	− 0.46	0.77	0.36	0.63 (0.14–2.87)	0.550
Empirical antibiotic treatment (within 1 month)					
Combination therapy	0.36	0.32	1.28	1.43 (0.77–2.67)	0.258
Length of antimicrobial therapy < 7 days	0.93	0.30	9.39	2.54 (1.40–4.60)	0.002*
Presence of indwelling catheters	− 0.16	0.29	0.31	0.85 (0.48–1.51)	0.576
Mechanical ventilation	1.43	0.55	6.78	4.17 (1.42–12.23)	0.009*
Septic shock	1.15	0.43	7.30	3.16 (1.37–7.30)	0.007*
Laboratory examination results					
Platelet count < 100.0 × 10^9^/L	0.19	0.34	0.31	1.21 (0.62–2.36)	0.578
Lymphocytes count < 1.0 × 10^9^/L	0.33	0.29	1.30	1.40 (0.79–2.48)	0.255
Albumin < 30.0 g/L	0.85	0.29	8.84	2.34 (1.34–4.10)	0.003*
Fungi					
* Candida albicans*	0.22	0.43	0.25	1.24 (0.53–2.90)	0.617
* Aspergillus flavus*	0.64	0.69	0.85	1.89 (0.49–7.32)	0.355

SE, standardized error; OR, odds ratio; ECOG-PS, Eastern Cooperative Oncology Group Performance Status; BSI, bloodstream infection; COPD, chronic obstructive pulmonary disease

^*^Indicates statistical significance

### Construction and validation of the nomogram

**Fig. 2:**
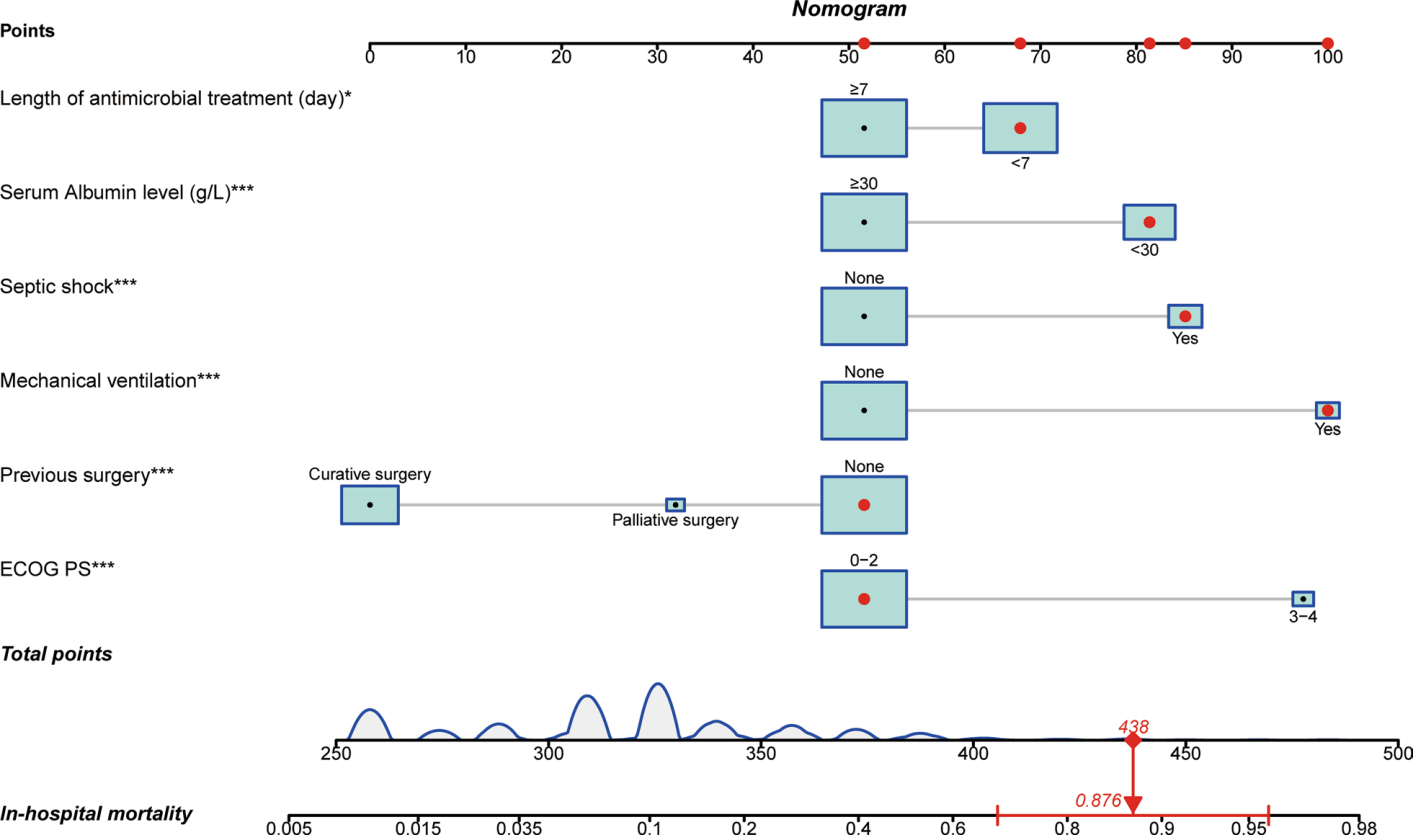
The constructed nomogram for predicting in-hospital death risk of nosocomial infections in cancer patients. This patient is a 65 years old female diagnosed with stage IV colorectal cancer (T4aN2bM1) and received bevacizumab plus irinotecan and S-1 for anti-tumor therapy. This patient was admitted to our hospital due to urgently occurred fever and chill and was diagnosed with BSI caused by ESBL-producing *E. coli* through a blood culture. Unfortunately, the patient eventually died of septic shock even an intense antimicrobial regimen was initiated. According to the nomogram, we can calculate that the total point for this patient is 438 and its corresponding in-hospital death risk is 87.6%

**Fig. 3:**
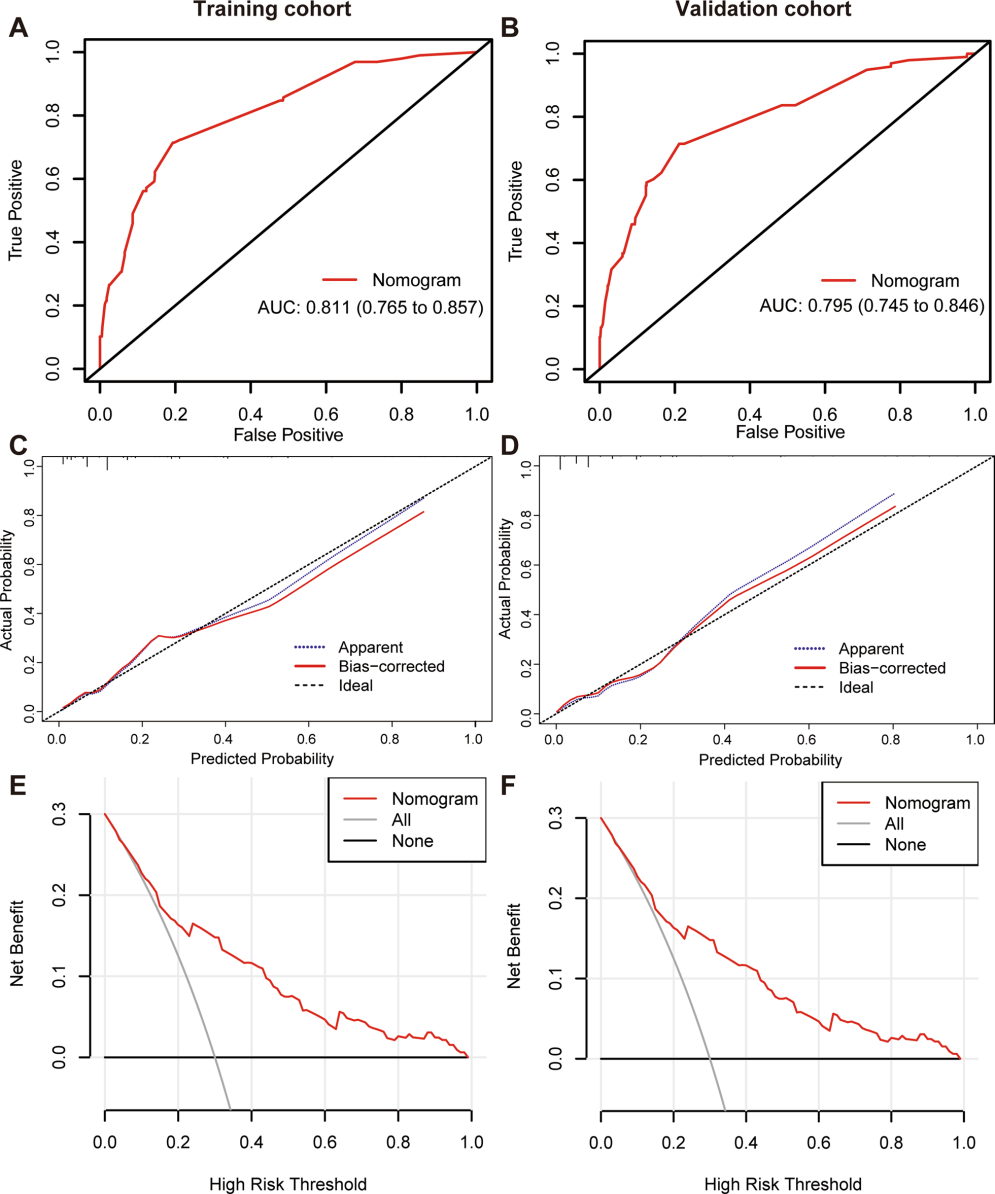
Assessment of the predictive ability and clinical utility of the nomogram for predicting in-hospital death risk of nosocomial infections in cancer patients. **A**,** B** The ROC curves of the nomogram for predicting in-hospital death risk of nosocomial infections in cancer patients in the training cohort (**A**) and validation cohort (**B**). **C**,** D** The calibration curves of the nomogram for predicting in-hospital death risk of nosocomial infections in cancer patients in the training cohort (**C**) and validation cohort (**D**). **E**,** F** Decision curve analysis of the nomogram for predicting in-hospital death risk of nosocomial infections in cancer patients in the training (**E**) and validation (**F**) cohorts. ROC, receiver operating characteristic curve

A nomogram was constructed based on the six independent predictive variables to estimate each patient's in-hospital mortality easily. The clinicians could evaluate the in-hospital death risk of cancer patients once they developed nosocomial infections during hospitalization. Furthermore, they could recognize high-risk patients and take appropriate measures to minimize the in-hospital death risk. Figure 2 gave an example to show how the nomogram could be used as an accurate prognostic stratification tool for nosocomial infections in cancer patients. Additionally, multiple tests were performed to assess the predictive ability and clinical utility of the nomogram in predicting the in-hospital death risk of nosocomial infections among oncological patients in the training and validation cohorts. We observed that the area under the curve (AUC) values of the nomogram were 0.811 (95% CI 0.765 to 0.857) and 0.795 (95% CI 0.745 to 0.846) in the training and validation cohorts (Fig. 3A, B). Besides, the calibration curves also revealed high consistencies between the actual and predicted in-hospital mortality in the two cohorts (Fig. 3C, D), suggesting that the constructed nomogram had good discrimination and calibration abilities. Given that the ROC curves and calibration curves are based on the specificity and sensitivity of the predictive model, they could not recognize “false positive” and “false negative” cases. DCA was conducted to evaluate the clinical utility and net clinical benefits of the nomogram when it was adopted to support clinical practice. We found higher clinical net benefits when the risk threshold was between 0 and 1.0 (Fig. 3E, F), indicating that the nomogram could significantly reduce the in-hospital death risk of nosocomial infections in cancer patients when used to guide clinical practice. Consistent results were also observed in clinical impact curves both in training and validation cohorts (Additional file 1: Fig. S3).

## Discussion

Patients with malignancy are more vulnerable to developing infections for various reasons (e.g., malnutrition, invasive procedures, surgery, immune suppression caused by cytotoxic treatment) [2]. Cancer patients have a bleak prognosis in nosocomial infections than noncancer patients, which means poor outcomes, prolonged hospitalization, more ICU admissions, extra medical costs, and higher mortality [3, 4, 16, 17]. Therefore, we conducted the current study to fully understand the clinical characteristics of nosocomial infections in patients with solid tumors and established a predictive model to predict the in-hospital mortality of these patients. This study systematically explored the clinical features of nosocomial infections in cancer patients, including microbiological distribution characteristics, infection-related features, and antimicrobial resistance patterns. Most importantly, we investigated the predictive factors for in-hospital death risk of nosocomial infections in cancer patients and developed and verified a nomogram that could accurately estimate the in-hospital death risk for each patient.

In the current study, 6.9% of patients with malignancy had nosocomial infections over the six years study period, which was low compared with the results of studies conducted in Ethiopia (19.4%) [6], Sudan (48.1%) [18], and India (31.33%) [19]. This discrepancy could be explained by the fact that the prevalence of hospital-acquired infections in cancer patients varies widely from region to region. In our study, we found that gram-negative bacteria were responsible for 63.3% of nosocomial infection episodes. In contrast, gram-positive cocci were responsible for 16.8% of cases, consistent with the results in other hospital settings [2, 20, 21]. However, several studies reported that gram-positive cocci represented the primary pathogens for nosocomial infections [6, 22–24]. This may be attributed to the population in these studies including hematological patients, and most of them combined with neutropenia. Furthermore, the epidemiology features of nosocomial infections in oncological patients have swerved from gram-positive cocci to gram-negative bacteria in the previous 20 years, as we saw worldwide [25]. These findings suggest that regular monitoring is necessary to understand the epidemiological characteristics of nosocomial infections among cancer patients in the local hospital.

In this study, the MDR phenomenon was observed in 25.5% of nosocomial infection cases, with MDRGNB being the most predominant causative pathogens, accounting for 24.8% of all infection episodes. Of these, ESBL-producing *E. coli* was the most common MDR pathogen. A similar result was also reported in a retrospective study conducted in Spain [26]. Recently, ESBL-producing *E. coli* has emerged as an important pathogen associated with nosocomial infections worldwide. ESBL has been a crucial mechanism of third-generation cephalosporin resistance, leading to a series of treatment problems [27]. Therefore, it is critical for cancer patients with nosocomial infections to initiate appropriate antibiotic treatment. Generally, the β-lactam + β-lactamase inhibitor is the most common empirical antibiotic treatment in cancer patients with hospital-acquired infections. However, the antimicrobial susceptibility of the isolated gram-negative bacteria showed that these pathogens exhibited extraordinary sensitivity to piperacillin/tazobactam, meropenem, imipenem, and amikacin, regardless of their ESBL status, which is in line with a study conducted in southern Taiwan [21]. Thus, piperacillin/tazobactam could serve as an initial empirical antibiotic regimen for cancer patients with nosocomial infections.

In the current study, 12.1% of cancer patients died from nosocomial infections, which is lower than the results of previous studies reported by Marin et al. (32%) [28] and Wisplinghoff et al. (35.5%) [24]. This difference can be explained by the fact that BSI was the most common infection type in these studies. Additionally, we investigated the predictive factors for in-hospital death risk of nosocomial infections among cancer patients. We identified that ECOG-PS 3–4, mechanical ventilation, septic shock, inadequate antimicrobial treatment, and hypoproteinemia were independent risk factors for in-hospital death risk. However, previous curative surgery was a protective factor for in-hospital mortality. Interestingly, Tu et al. [29] reported that intra-abdominal infection did not cause reduced long-term survival of patients who went through curative surgery. On the one hand, it may be attributed to the fact that patients who received curative surgery had no distant metastasis. On the other hand, considering only a small number of patients were enrolled in this subgroup, it needs to be further validated before drawing the conclusion.

Generally speaking, cancer patients with worse ECOG-PS were correlated with unfavorable survival outcomes. In this study, we observed that patients with worse ECOG-PS had higher in-hospital mortality when these patients developed nosocomial infections. It suggests that we should pay close attention to these populations. Besides, appropriate and precise management should be initiated rapidly once infection onsets. We also found that patients who received mechanical ventilation and inadequate antimicrobial therapy during hospitalization were associated with a worse prognosis, which was compatible with previous studies [11, 20, 29–31]. Septic shock was identified as the most predominant risk factor for a worse survival probability of nosocomial infections [11, 20, 32]. As we know, patients with septic shock or who received mechanical ventilation always combined with circulatory and/ or respiratory dysfunction, resulting in poor clinical outcomes. Furthermore, we observed that patients with hypoproteinemia were significantly related to higher in-hospital mortality. Previous studies reported that hypoproteinemia was frequently related to reduced quality of life and diminished life expectancy due to immunosuppression and diminished muscle mass in patients with malignancy [33, 34]. In addition, Paccagnella A et al. also reported that hypoproteinemia in cancer patients could result in malnutrition and weight loss, leading to a poor prognosis and increased cancer-associated deaths in these patients [35, 36]. Taken together, clinicians should focus more on the respiratory and circulatory conditions of cancer patients once they developed severe nosocomial infections. Besides, it also suggested that rapid recognition of septic shock, early and effective empirical antibiotics treatment, monitoring of nutritional status, and supportive care are also crucial in improving the prognosis of these patients.

Currently, nomogram is widely used in the research of cancer and other fields. The nomogram can transform the sophisticated regression equation into an intuitive graph, making the patients’ prediction risk readable. In the present study, we developed a nomogram to predict the in-hospital death risk of nosocomial infections in cancer patients. We also evaluated its predictive ability and clinical utility when it was adopted to support decision-making in practice. Altogether, the nomogram has good performance in predicting the in-hospital death risk of these individuals. To the best of our knowledge, this is the first study that systematically evaluated the clinical features of nosocomial infections among cancer patients in Northwestern China. Most importantly, we developed and verified a nomogram that could accurately predict the in-hospital death risk of nosocomial infections among these patients. Nevertheless, our study also has several inevitable shortcomings. First, it is challenging to collect some variables (such as concrete chemotherapeutic and radioactivity dosage, concrete antibiotics treatment information before admission, and some detailed laboratory examination results) because of the design of retrospective analysis. Thus, potential biases might exist in this study. Second, although we established a nomogram that could effectively predict the in-hospital death risk of nosocomial infections in patients with solid tumors and validated its predictive ability in a validation cohort, lacking an independent external validation cohort is a disadvantage for this study. Therefore, multicenter retrospective and well-designed prospective studies are urgently needed in the future to verify the performance of the nomogram.

## Conclusions

In conclusion, nosocomial infections are common in oncological patients. Gram-negative bacteria are still the most frequently isolated pathogens. MDR phenomenon is not rare for nosocomial infections in these patients, with MDRGNB being the most predominant MDR strain. Besides, we constructed and validated a novel nomogram that could accurately predict the in-hospital death risk of nosocomial infections in cancer patients. Precise management of high-risk patients, early recognition of septic shock, rapid and adequate antimicrobial treatment, and dynamic monitoring of serum albumin levels may improve the prognosis of these individuals.

## Supplementary Information

**Additional file 1: Fig. S1**. Microbiological distribution characteristics of nosocomial infections in cancer patients. (**A**) Microbiological distribution characteristics between different medical wards. (**B**) Microbiological distribution characteristics between different cancer types. **Fig. S2**. Antimicrobial resistance patterns of nosocomial infections in cancer patients. (**A**) Antimicrobial resistance pattern of gram-negative bacilli. (**B**) Antimicrobial resistance pattern of gram-positive cocci. TZP, Piperacillin/tazobactam; SXT, Sulfamethoxazole-trimethoprim. **Fig. S3**. Clinical impact curves of the nomogram for predicting in-hospital death risk of nosocomial infections in cancer patients in the training (**A**) and validation (**B**) cohorts.

## Data Availability

The data can be available upon reasonable request to the corresponding authors after and approval from the First Affiliated Hospital of Xi’an Jiaotong University Committee of Ethics.

## Abbreviations

RDI: Relative dosage intensity
BSIs: Bloodstream infections
MDR: Multidrug-resistant
ECOG-PS: Eastern Cooperative Oncology Group performance status
ICU: Intensive care unit
BALF: Bronchoalveolar lavage fluid
CLSI: Clinical and Laboratory Standards Institute
SD: Standard deviations
IQR: Interquartile
ROC: Receiver operating characteristic
DCA: Decision curve analysis
COPD: Chronic obstructive pulmonary disease
MDRGNB: Multi-drug resistant gram-negative bacilli
ESBL: Extended-spectrum β-lactamase
OR: Odds ratio
CI: Confidence interval
AUC: Area under the curve
TZP: Piperacillin/tazobactam
SXT: Sulfamethoxazole-trimethoprim
