# Supplementary figures and images for: Establishment and validation of a nomogram to predict the in-hospital death risk of nosocomial infections in cancer patients
Source: Antimicrob Resist Infect Control. 2022 Feb 7;11:29. doi: 10.1186/s13756-022-01073-3 (PMC8822816; doi:10.1186/s13756-022-01073-3)

Figure S1


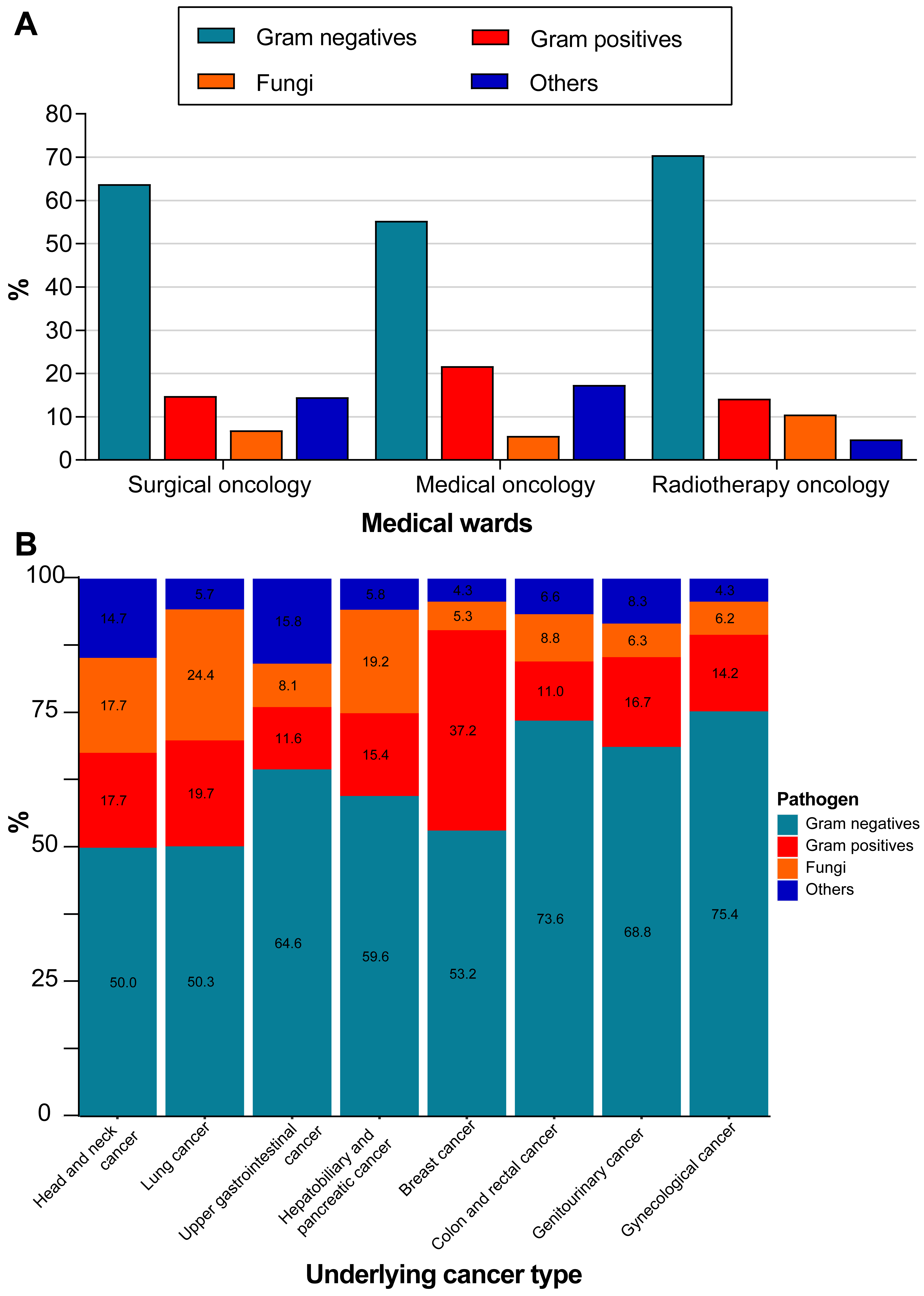


Figure S2


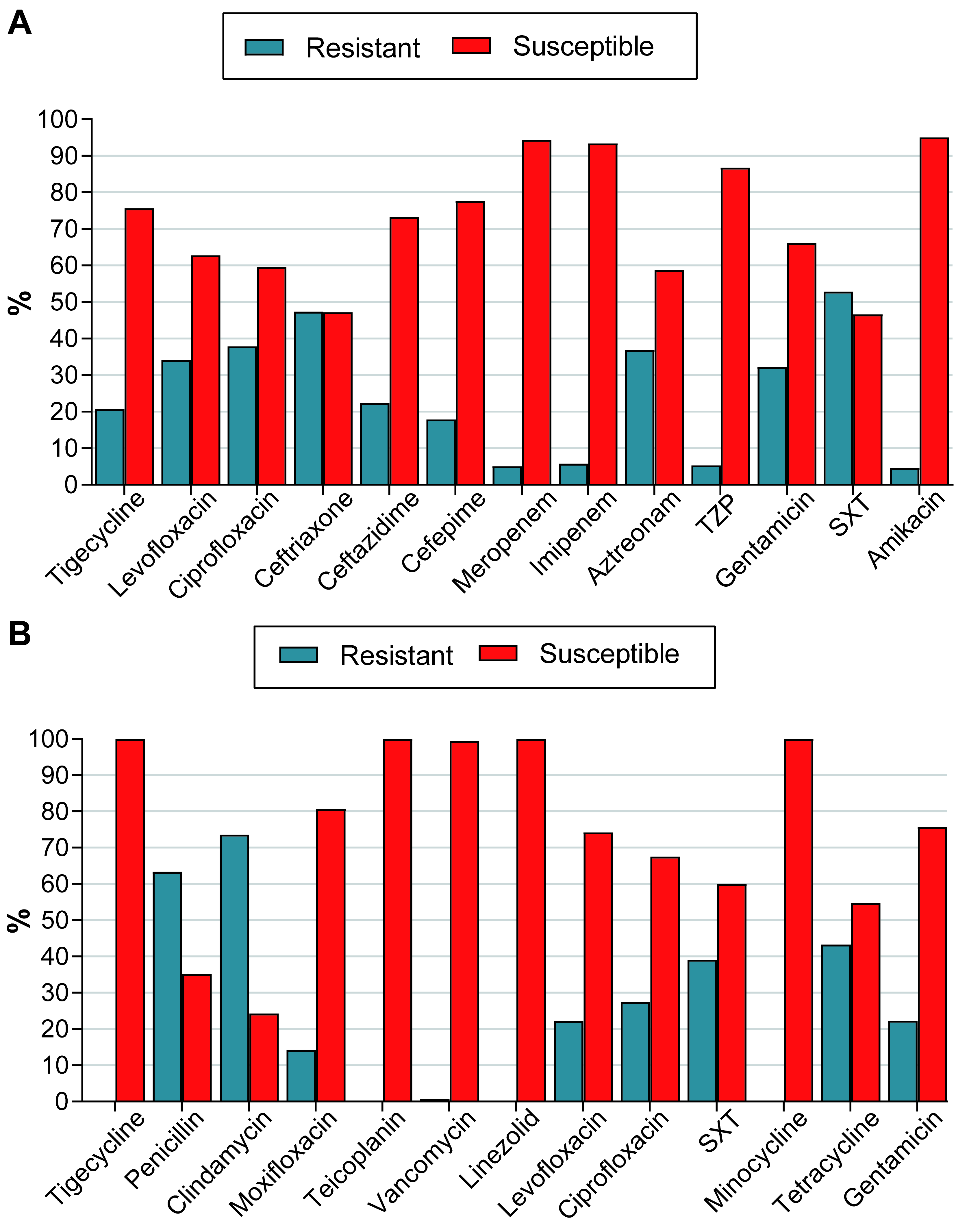


Figure S3


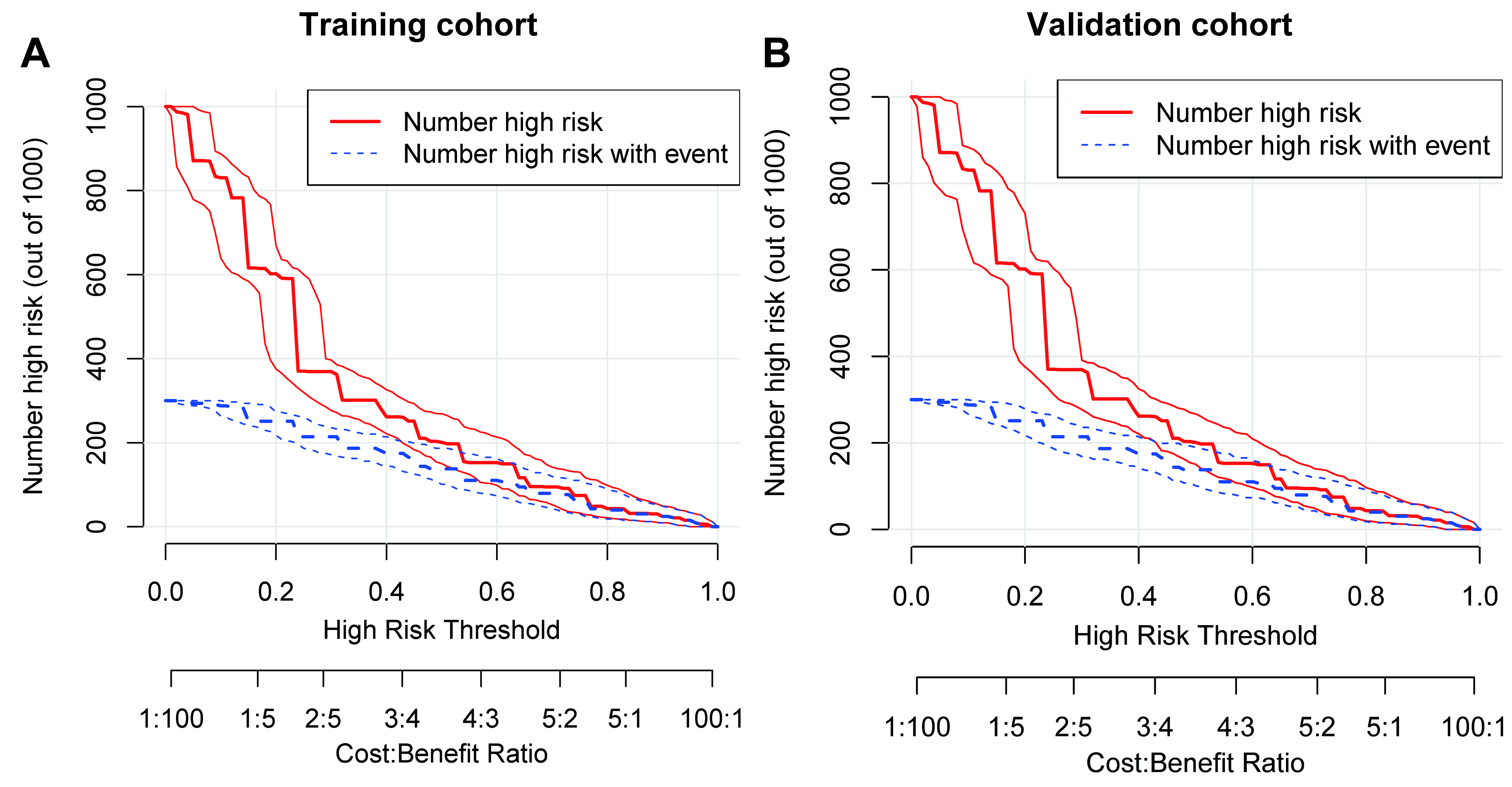

Supplement: Supplementary file 1 — Additional file 1: Fig. S1. Microbiological distribution characteristics of nosocomial infections in cancer patients. (A) Microbiological distribution characteristics between different medical wards. (B) Microbiological distribution characteristics between different cancer types. Fig. S2. Antimicrobial resistance patterns of nosocomial infections in cancer patients. (A) Antimicrobial resistance pattern of gram-negative bacilli. (B) Antimicrobial resistance pattern of gram-positive cocci. TZP, Piperacillin/tazobactam; SXT, Sulfamethoxazole-trimethoprim. Fig. S3. Clinical impact curves of the nomogram for predicting in-hospital death risk of nosocomial infections in cancer patients in the training (A) and validation (B) cohorts. [file 13756_2022_1073_MOESM1_ESM.docx]
